# Transcriptional Changes after Enniatins A, A1, B and B1 Ingestion in Rat Stomach, Liver, Kidney and Lower Intestine

**DOI:** 10.3390/foods10071630

**Published:** 2021-07-14

**Authors:** Alessandra Cimbalo, Manuel Alonso-Garrido, Guillermina Font, Massimo Frangiamone, Lara Manyes

**Affiliations:** Laboratory of Food Chemistry and Toxicology, Faculty of Pharmacy, University of Valencia, Avenue Vicent Andrés Estellés s/n, 46100 Burjassot, Spain; alscim@uv.es (A.C.); manuel.alonso-garrido@uv.es (M.A.-G.); masfran4@uv.es (M.F.); lara.manyes@uv.es (L.M.)

**Keywords:** enniatins, oxidative phosphorylation, in vivo, quantitative Real-Time PCR (qPCR)

## Abstract

Enniatins (ENs) are depsipeptide mycotoxins produced by *Fusarium* fungi. They are known for their capacity to modulate cell membrane permeability and disruption of ionic gradients, affecting cell homeostasis and initiating oxidative stress mechanisms. The effect of the acute toxicity of ENs A, A1, B and B1 at two different concentrations after 8 h of exposure was analysed in Wistar rats by a transcriptional approach. The following key mitochondrial and nuclear codified genes related to the electron transport chain were considered for gene expression analysis in stomach, liver, kidney and lower intestine by quantitative Real-Time PCR: mitochondrially encoded NADH dehydrogenase 1 (MT-ND1), mitochondrially encoded cytochrome c oxidase 1 (MT-COX1), succinate dehydrogenase flavoprotein subunit A and ATP synthase F1 subunit alpha, respectively. Moreover, the expression of markers involved in oxidative stresssuperoxide dismutase 1 (SOD1), glutathione peroxidase 1 (Gpx1), heme oxygenase 1, apoptosis B-cell lymphoma 2, Bcl2 Associated protein X (Bax), tumor suppressor protein (p53), inhibition of apoptosis nuclear factor kappa of activated B cells, immune system interleukin 1β and intestinal tight junction Occludin merely in lower intestine tissues have been investigated. For mitochondrial genes, the main differences were observed for MT-ND1 and MT-COX1, showing its deficiency in all selected organs. With regard to the intestinal barrier’s cellular response to oxidative stress, the activity of the antioxidant gene SOD1 was decreased in a dose-dependent manner. Similarly, the catalytic enzyme GPx1 was also downregulated though merely at medium dose employed. On the contrary, the pro-apoptotic Bax and p53 regulators were activated after ENs exposure, reporting a significant increase in their expression. Furthermore, the alteration of intestinal permeability was assessed by the abnormal activity of the tight junction protein occludin. In summary, ENs may generate mitochondrial disorders and induce oxidative stress in intestinal barrier function.

## 1. Introduction

In spite of many years of research and the introduction of good practices in the food production, storage and distribution chain, nowadays, mycotoxins are a big risk to food safety. A useful tool capable to protect consumers against their toxic effects is the hazard analysis and critical control points (HACCP) for production and storage, but currently this system only applies to legislated mycotoxins [1]. In the past, emerging mycotoxins have been considered less important because of their low probability of acute toxicity. Nevertheless, they have a high prevalence in food products, sometimes even in high concentrations [2].

Regarding the toxicity of enniatins (ENs), limited data was available until now, hence they are currently under occurrence and toxicity evaluation [3]. ENs are emerging mycotoxins produced by filamentous fungi of the *Fusarium* genus, mainly by the F. *acumuniatum*, F. *avenaceum*, *F. oxysporum, F. Poae, F. sporothrichioides, F. Sambucinum* and *F. Tricinctum* species [4]. They can be found in a wide variety of food and feed, mainly in cereals and their derivatives, dried fruit, spices, cocoa and coffee. ENs’ chemical structure corresponds to a cyclic depsipeptide, and so far, more than 23 belonging to the A, B and J types, have been identified [5]. However, the most frequent ENs detected in food and feed are enniatin A (ENA), enniatin A1 (ENA1), enniatin B (ENB) and enniatin B1 (ENB1) [6]. 

The toxicity of ENs is based on their ionophoric properties. They facilitate the transport of mono or divalent cations such as K^+^ or Ca^2+^ across membranes, thereby disrupting normal physiological concentrations of these ions [7]. Moreover, it has been shown that ENs can induce a cytotoxic effect by producing reactive oxygen species (ROS) and subsequently causing lipid peroxidation and alteration of the normal cell cycle due to their anti-proliferative effects on several cell types [5]. Furthermore, they can decrease the calcium retention capacity of the mitochondrion matrix leading to the collapse of the mitochondrial membrane potential via the permeability transition pore opening, and they can cause oxidative phosphorylation decoupling [8].

The European Food Safety Authority reported an ENB-induced genotoxic effect in vivo after acute oral administration [9]. Recently, the in vivo toxicity of ENs was reviewed, reporting immunotoxicity in peripheral blood lymphocytes in Wistar rats. ENB was found in high concentration in rats’ liver and fat, demonstrating the molecule’s tendency to bioaccumulate in lipophilic tissues. The jejunum, duodenum and colon were also identified as a possible absorption area for ENA in female rats during a sub-chronic exposure [10].

In vitro studies have confirmed ENs’ toxicity in several cell lines. ENB alone, or in mixture with other ENs, induced lysosomal disruption and necrosis in Caco-2 cells, lysosomal damage in mouse monocyte macrophage RAW 267.4 and apoptosis in rat hepatoma cell line H4IIE [11]. Likewise, ENs induced nuclear fragmentation and apoptotic body formation in adenocarcinomic human alveolar basal epithelial (A549), Small Cell Lung Carcinoma (GLC-4), human cervix carcinoma (KB-3-1) and human leukemia (HL-60) cells [12]. Moreover, the linkage between ENs toxicity and the alteration of mitochondrial related pathways has been recently investigated in vitro. Transcriptomics helped to observe how mitochondria were the main affected organelles in Jurkat cells after ENB exposure, finding the highest expression alterations in oxidative phosphorylation related genes [13]. ENs’ toxicity in human epithelial cells (ECV304) was also confirmed in the expression of several genes belonged to complex I (CI), IV and V, although ATP synthase resulted as the most affected [14]. Furthermore, after ENB and BEA mixture exposure, a proteomics analysis revealed changes in the protein levels in the inner and outer membrane in Jurkat cells in a concentration-dependent manner [15]. 

In order to better understand ENs’ toxicity, Wistar rats were chosen as an in vivo model to analyze changes in the expression of selected genes involved in electron transport chain (ETC), oxidative stress, apoptosis, inflammation and intestinal tight junction in rat stomach, liver, kidney and lower intestine. More specifically, mitochondrially encoded hydride nicotinamide adenine dinucleotide (NADH) dehydrogenase 1 (MT-ND1) belonging to CI, succinate dehydrogenase flavoprotein subunit A (Sdha) of complex II (CII), mitochondrially encoded cytochrome c oxidase 1 (MT-COX1) of CIV and ATP synthase F1 subunit alpha (ATP5) of CV activity were assessed in all the organs considered. Furthermore, the oxidative stress markers superoxide dismutase 1 (SOD1), glutathione peroxidase 1 (GPx1), heme oxygenase 1 (Hmox1), apoptosis regulators B-cell lymphoma 2 (Bcl2), Bcl2 Associated protein X (Bax), tumor suppressor protein p53, nuclear factor kappa of activated B cells (Nf-κB) inflammation cytokine interleukin 1β (Il-1β and intestinal tight junction protein Occludin were analyzed in colon tissues. 

## 2. Materials and Methods

### 2.1. Reagents

The standards of standard solution stock (purity: 99%) of ENA (mw: 681.92 g/mol), ENB (mw: 639.82 g/mol), ENA1 (mw: 667.87 g/mol), ENB1 (mw: 653.85 g/mol) and phosphate buffer saline (PBS) were obtained from Sigma-Aldrich (Madrid, Spain). All the stock solutions were prepared by dissolving 1 mg of mycotoxin in 1 mL of pure methanol, obtaining a 1 mg/mL (1000 mg/L) solution. These stock solutions were diluted with methanol in order to obtain the appropriate multi-compounds working standard solutions. All the standards were kept at −20 °C. 

For RNA extraction, TRIzol^TM^ reagent was purchased from Invitrogen™ (Carlsbad, CA, USA), whereas for its purification, the ReliaPrep™ RNA Miniprep System kit from Promega (Madison, WI, USA) was employed. Deionized water (resistivity < 18 MV cm) was obtained using a Milli-Q water purification system (Millipore, Bedford, MA, USA). TaqMan™ Reverse Transcription kit and PowerUp™ SYBR™ Green for quantitative Real-Time PCR (qPCR) analysis were purchased from Applied Biosystems (Carlsbad, CA, USA). 

### 2.2. In Vivo Study Design

Fourteen female Wistar rats (243–278 g) were acquired from Pharmacy animal facility (Universitat de València, Valencia, Spain). Animals were divided in three groups: four rats in the control group and five rats in each treated one, with medium and high exposure. Each group was housed in one cage in a windowless room with a 12-h light–dark cycle. The study rooms were maintained under controlled conditions appropriate for the species (temperature 22 °C, relative humidity 45–65%). The control group was exposed to the vehicle (PBS), while five of the treated ones were intoxicated with medium concentrations: single dose of EN A 256, ENA1 353, ENB 540, ENB1 296 μg/mL; and the other five were intoxicated with the higher ones: single dose of ENA 513, ENA1 706, ENB 1021, ENB1 593 μg/mL. Mycotoxins were administered dissolved by sonication in 1 mL of PBS by oropharyngeal administration using a metal cannula. After 8 h exposure with water but no feed, they were sacrificed by isoflurane gas asphyxiation and the organs (liver, stomach, kidneys, lower intestine) were removed and stored at −20 °C.

### 2.3. RNA Extraction

Total RNA of the control and exposed rats was isolated using from approximately 50 mg of frozen tissue according to TRIzol^TM^ manufacturer’s protocol (Invitrogen™,Carlsbad, CA, USA). Samples were homogenized in TRIzol^TM^ (50 mg/mL) with a T25 Ultra-turrax Digital High-Speed Homogenizer (IKA^®^, Staufen, Germany). Extracted RNA was purified according to ReliaPrep™ RNA Miniprep System kit (Promega, Madison, WI, USA). The purity and quantity of RNA were evaluated spectrophotometrically using a NanoDrop™ 2000 (Thermo Scientific™, Madrid, Spain), showing concentrations between 370 and 2359 ng/μL and appropriate 260/280 nm and 260/230 nm ratios both around 2 (Table 1). RNA samples were stored until their dilution to 100 ng/μL with ultrapure Milli QH_2_O system until their reverse transcription to cDNA.

### 2.4. Gene Selection and Primer Design

Gene-specific primers were designed using Primer-BLAST by using default criterion of the software with amplified products ranging from 83 to 122 bp and Tm at 58 °C. Primer sequences were used in qPCR analyses. Standard curve by qPCR was performed for all primer’s pairs and a single amplification product for each gene was obtained by the melting curve assay StepOne Plus Real-time PCR instrument (Applied Biosystems, Foster city, CA, USA). Primer amplification efficiency was determined from standard curve generated by serial dilution of cDNA (5-fold each) for each gene. Correlation coefficient (R2 values) and amplification efficiencies (E) for each primer pairs were calculated from slope of regression line by plotting mean Cq values against the log cDNA dilution factor in StepOne software (Table 2). 

### 2.5. Reverse Transcription and qPCR

Real-time amplification reactions were performed in 96 well plates using SYBR Green detection chemistry and were run in triplicate on 96-wells plates with the StepOne Plus Real-time PCR instrument (Applied Biosystems, Foster City, CA, USA). Reactions were prepared as follows: 100 ng template, 500 μM of each primer, the required amount of 2x Fast SYBR Green and completed to 20 μL with RNAse free water (Applied Biosystems, Foster City, CA, USA). The cycling conditions were set as default: initial denaturation step of 95 °C for 5 min to activate the Taq DNA polymerase, followed by 40 cycles of denaturation at 95 °C for 15 s, annealing at 58 °C for 15 s and elongation at 72 °C for 30 s. The melting curve was generated by heating the amplicon from 60 to 90 °C. Therefore, threshold cycles (Ct) were automatically determined using the StepOne Plus Software version 2.3 (Applied Biosystems, Foster, CA, USA). Three technical replicates were performed for each condition. Experiments were performed according to MIQE (Minimum Information for Publication of Quantitative Real-Time PCR Experiments) guidelines [16].

### 2.6. Statyistical Analysis

Normalized Cp were calculated per sample as ΔCt (experimental Ct—housekeeping Ct mean) by using Ct values obtained by qPCR. A t-Student test was applied to evaluate differences between each mycotoxin exposed sample group and the control considering *p* ≤ 0.05 as statistically significant. Statistical analysis was assessed by SPSS 24.0 (IBM Corp., Armonk, NY, USA). For gene expression analysis, three technical replicates of each sample were analyzed for control group (C1, C2, C3, C4), medium dose treated group (M1, M2, M3, M4, M5) and high dose treated group (H1, H2, H3, H4, H5). Log2RQ median of all genes was calculated for each condition, considering C1 as Log2RQ = 0. 

## 3. Results

### 3.1. Activity of Mitochondrial and Nuclear Encoded Genes in Stomach

**Figure 1:**
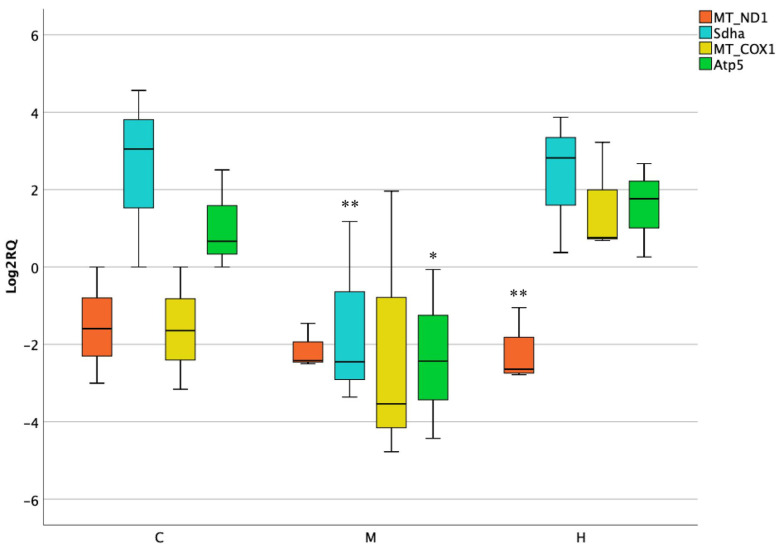
Box plot showing relative expression of mitochondrial and nuclear encoded genes in stomach when compared to control C1 (Log2RQ = 0) to medium treatment (M) and high treatment (H) by quantitative PCR (qPCR). RQ, relative quantification. The box plots show the median value (horizontal line), the top and bottom edges of the boxes (75% and 25% percentiles) and whiskers, which are the furthest values away from the boxes that are not considered outliers. * *p* < 0.05; ** *p* < 0.01 significantly different from the control.

The transcriptional analysis of the selected mitochondrial genes was performed by qPCR technique. In the first organ studied, a remarkable downregulation of mitochondrial encoded MT-ND1 at highest dose was observed when compared to the control. Likewise, nuclear encoded genes Sdha and Atp5 resulted as downregulated but solely after medium treatment (Log2RQ = −3.5) (Figure 1). 

### 3.2. Activity of Mitochondrial and Nuclear Encoded Genes in Liver

**Figure 2:**
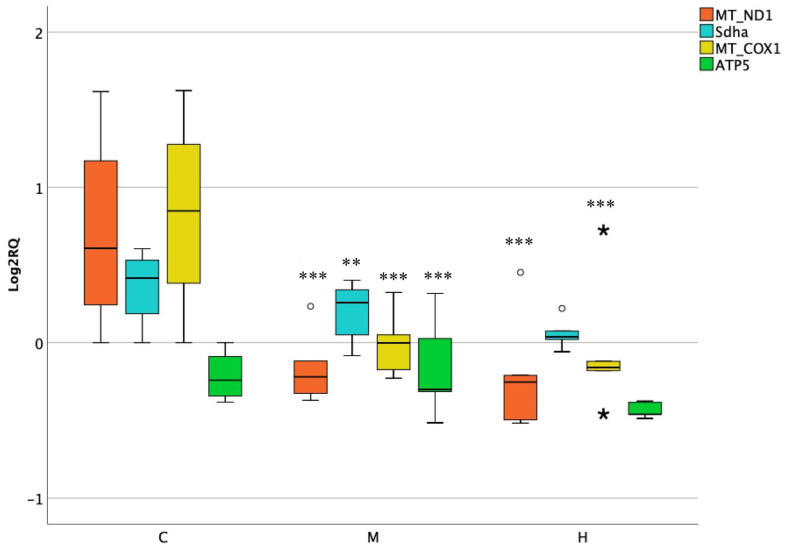
Box plot showing relative expression of mitochondrial and nuclear encoded genes in liver when compared to control C1 (Log2RQ = 0) to medium treatment (M) and high treatment (H) by qPCR. RQ, relative quantification. The box plots show the median value (horizontal line), the top and bottom edges of the boxes (75% and 25% percentiles), (★) extreme cases, (o) outliers (atypical values) and whiskers, which are the furthest values away from the boxes that are not considered outliers. ** *p* < 0.01; *** *p* < 0.001 significantly different from the control.

The hepatic investigation after acute exposure to ENs reported significant changes of all genes analyzed when treated with a medium dose. Once administering higher dose, merely MT-ND1 and MT-COX1 revealed a considerable decrease of roughly twofold compared to the control (*p* = 0.0002). Nevertheless, a moderate interindividual variability was observed in the control groups of the latter genes (Figure 2).

### 3.3. Activity of Mitochondrial and Nuclear Encoded Genes in Kidneys

**Figure 3:**
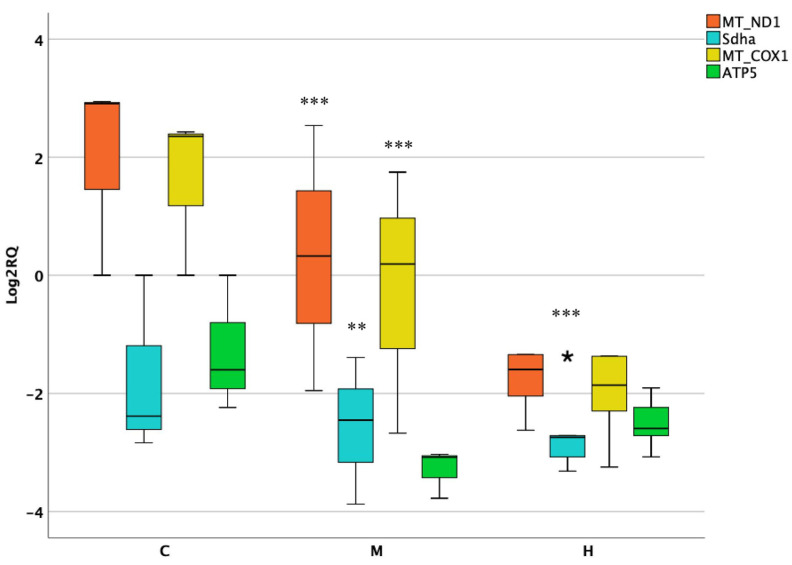
Box plot showing relative expression of mitochondrial and nuclear encoded genes in kidney when compared to control C1 (Log2RQ = 0) to medium treatment (M) and high treatment (H) by qPCR. RQ, relative quantification. The box plots show the median value (horizontal line), the top and bottom edges of the boxes (75% and 25% percentiles), (★) extreme cases and whiskers, which are the furthest values away from the boxes that are not considered outliers. ** *p* < 0.01; *** *p* < 0.001 significantly different from the control.

Regarding the kidneys, the expression of MT-ND1 and MT-COX1 was strongly affected at medium dose. A significant downregulation of both genes was observed (*p* = 0.0001), although showing a noticeable interindividual variability among samples. Similarly, Sdha followed the same trend, but in this case, a downregulation of nearly fourfold was found in a dose-dependent manner (Figure 3).

### 3.4. Activity of Mitochondrial and Nuclear Encoded Genes in Lower Intestine

**Figure 4:**
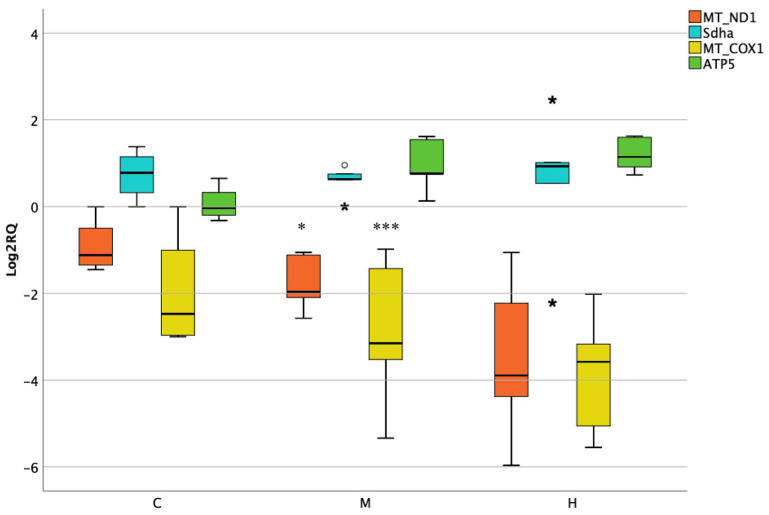
Box plot showing relative expression of mitochondrial and nuclear encoded genes in lower intestine when compared to control C1 (Log2RQ = 0) to medium treatment (M) and high treatment (H) by qPCR. RQ, relative quantification. The box plots show the median value (horizontal line), the top and bottom edges of the boxes (75% and 25% percentiles), (★) extreme case and whiskers, which are the furthest values away from the boxes that are not considered outliers. * *p* < 0.05; *** *p* < 0.001 significantly different from the control.

When studying the rats’ colon tissues, MT-ND1 and MT-COX1 were significantly downregulated at medium dose, but not at the higher one. On the contrary, nonsignificant changes were observed for nuclear encoded genes (Figure 4). 

### 3.5. Activity of Oxidative Stress Genes in Lower Intestine 

**Figure 5:**
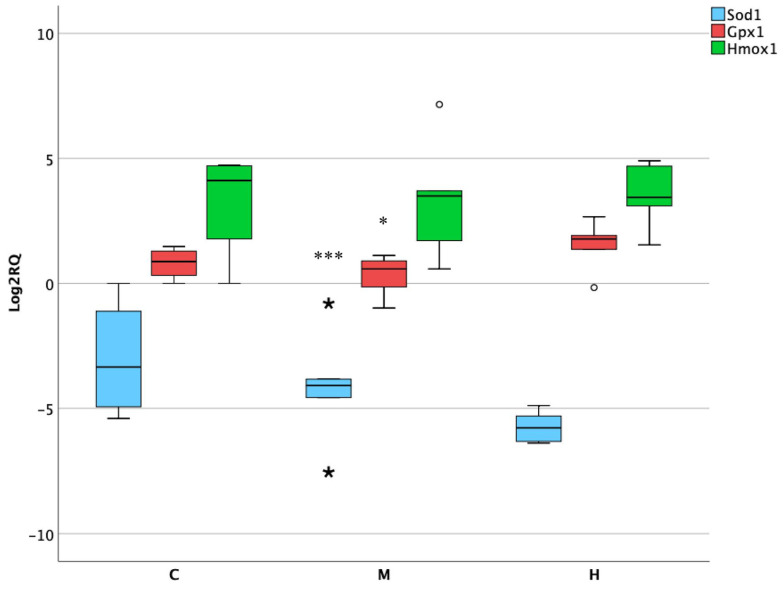
Box plot showing relative expression of oxidative stress genes in lower intestine when compared to control C1 (Log2RQ = 0) to medium treatment (M) and high treatment (H) by qPCR. RQ, relative quantification. The box plots show the median value (horizontal line), the top and bottom edges of the boxes (75% and 25% percentiles), (★) extreme cases, (o) outliers (atypical values) and whiskers, which are the furthest values away from the boxes that are not considered outliers. * *p* < 0.05; *** *p* < 0.001 significantly different from the control.

The intestinal tract is frequently exposed to ROS production and the associated activation antioxidant enzyme defense systems. For this purpose, genes involved in oxidative stress processes were selected. After the treatments, the most altered was SOD1, showing a loss of its expression, which was significant at medium dose (*p* = 0.0002). In addition, the antioxidant enzyme GPx1 was slightly downregulated but had a weaker response to the medium treatment (*p* = 0.05) (Figure 5). 

### 3.6. Activity of Apoptotic Genes in Lower Intestine

**Figure 6:**
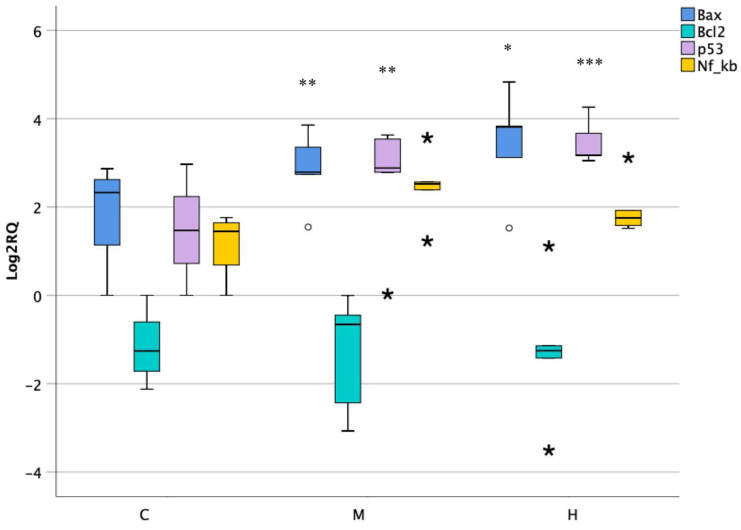
Box plot showing relative expression of apoptotic genes in lower intestine when compared to control C1 (Log2RQ = 0) to medium treatment (M) and high treatment (H) by qPCR. RQ, relative quantification. The box plots show the median value (horizontal line), the top and bottom edges of the boxes (75% and 25% percentiles), (★) extreme cases, (o) outliers (atypical values) and whiskers, which are the furthest values away from the boxes that are not considered outliers. * *p* < 0.05; ** *p* < 0.01; *** *p* < 0.001 significantly different from the control.

Apoptosis is an important process which maintains the function of the intestinal barrier at its normal state by regulating its homeostasis. In this study, the activity of genes implicated in apoptotic pathways was evaluated. The expression of pro-apoptotic genes Bax and p53 showed an overexpression of nearly fourfold changes at both doses. On the contrary, the nuclear factor Nf-κB and the anti-apoptotic gene Bcl2 did not report significant changes when compared to the control (Figure 6). 

### 3.7. Inflammatory Response and Permeability in Lower Intestine 

**Figure 7:**
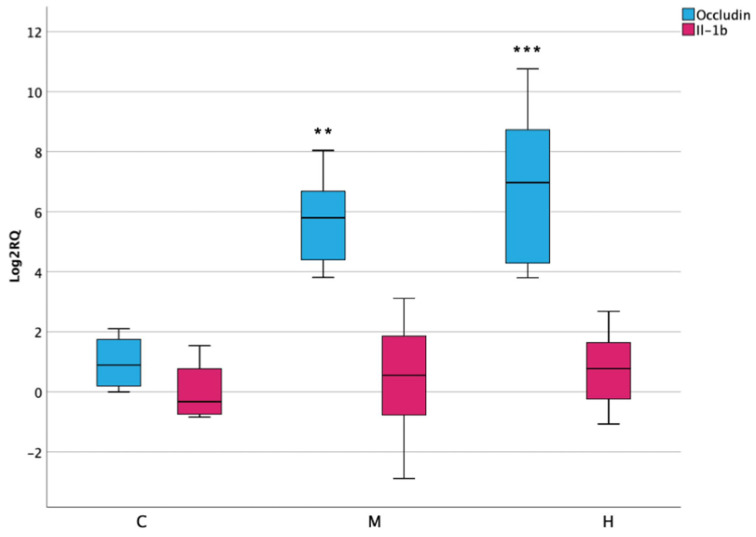
Box plot showing relative expression of Il-1β in lower intestine when compared to control C1 (Log2RQ = 0) to medium treatment (M) and high treatment (H) by qPCR. RQ, relative quantification. The box plots show the median value (horizontal line), the top and bottom edges of the boxes (75% and 25% percentiles) and whiskers, which are the furthest values away from the boxes that are not considered outliers. ** *p* < 0.01; *** *p* < 0.001 significantly different from the control.

The activation of oxidative stress and apoptotic processes was checked by the activity of pro-inflammatory cytokine Il-1β, which surprisingly did not report significant alterations after ENs exposure. Moreover, the function of intestinal permeability was evaluated by assessing the expression of occludin, an integral membrane protein localized at intestinal epithelial tight junction barrier. In this case, results have shown a significant increase in its expression in both medium and high treatment, reaching up to 10-fold (Figure 7).

## 4. Discussion

A recent proteomics approach confirmed the toxic capacity of ENs after acute exposure in rat’s liver and pointed to diverse biological processes as a target [17]. Proteomics findings showed that 13 of the significantly altered proteins were involved in the mitochondria, among which are ATP5 alpha and beta. ATP5 alpha protein expression was enhanced at both doses (log2FC medium dose: 4.1144; high dose: 2.2354) as well as antioxidant activity, showing the upregulation of four proteins including the SOD1 enzyme, but solely at the highest exposure (log2FC high dose:9.6712). In this study, using those liver samples and adding the stomach, kidney and lower intestine from the same animals, changes in the expression of selected genes related to mitochondria, oxidative stress, apoptosis, inflammatory response and permeability processes were evaluated. These findings have shown that acute exposure to ENs caused changes in the expression of all analyzed genes depending on the type of tissue.

Regarding the gene expression in the ETC, the first complex, mitochondrial CI deficiency is the most prevalent defect in the respiratory chain causing mitochondrial disorders. The activity of CI subunit 1 MT-ND1 is involved in the formation of the fourth proton pumping site by promoting proton translocation across the membrane [18]. After ENs administration, it has been observed a leakage of its expression in all organs analyzed, which could be related to an impairment of membrane homeostasis. According to these findings, lower activity of this gene was implicated in non-alcoholic fatty acid liver disease in rats and chronic kidney disease in mice [19,20]. However, on the contrary, elevated levels of MT-ND1 expression indicated mitochondrial dysfunction in the villous adenoma of human tissue, resulting with the accumulation of mutations in mtDNA [21]. 

Secondly, succinate-coenzyme Q reductase is a mitochondrial enzyme complex consisting of four protein subunits (Sdha, Sdhb, Sdhc and Sdhd), which is involved in the tricarboxylic acid cycle (TCA) and the ETC [22]. The loss of the normal TCA cycle promotes tumorigenesis due to metabolic alterations with enforced dependence on glycolysis for energy production [23]. CII Subunit -a was altered by ENs mixture, being downregulated in rats’ stomach, liver and kidneys, hinting at an impairment caused by these mycotoxins. According to previous findings, the lack of this gene is associated with mitochondrial dysfunction, which led to the development of wild type gastrointestinal stromal tumors and hereditary renal cell carcinoma [24,25]. 

Thirdly, cytochrome *c* oxidase, localized in the inner mitochondrial membrane, is the final electron acceptor in the ETC and its deficiency is a prevalent cause of oxidative stress status in mitochondria [26]. MT-COX1 gene is the main subunit of the complex, and it is responsible for the homeostatic synthesis of prostanoids [27]. The expression of MT-COX1 was significantly decreased in the liver at both doses of ENs employed, along with renal and intestinal tissues, although solely at medium dose. Its downregulation is related to the progression of esophageal adenocarcinoma, colon carcinogenesis, reduced proliferation and increased macro autophagy [28,29]. Moreover, MT-COX1 deficiency exacerbates hepatic diseases in mice [30] as well as in the present research, suggesting that ENs’ toxicity could play a role in hepatic function. Contrasting with this evidence, MT-COX1 resulted as overexpressed in mammalian and zebrafish kidneys under oxidative stress conditions [31,32]. 

The last complex studied was mitochondrial ATP synthase (CV), which is formed of two functional domains: F_1_, situated in the mitochondrial matrix and F_o_, located in the inner mitochondrial membrane. It produces most of the cell ATP by rotary catalysis, and its deficiency plays a crucial role in severe human disorders such as neuropathy, ataxia, encephalopathy (Leigh syndrome) and hypertrophic cardiomyopathy [33,34]. ATP5, the subunit α of CV F_1_ domain, was downregulated in gastric and hepatic tissues, but solely after medium treatment. According with these findings, reduced levels of its expression led to the decrease of oxidative phosphorylation in chicks, prostate and lung cancer, decrease in tissue metabolism, reduction in protein synthetic capacity and impairment of ATP-biosynthetic functions in rat liver [35,36]. In contrast, Ref. [37] reported highly overexpressed ATP5 in glioblastoma tumor cells and endothelial cells of microvascular proliferation.

Beyond the mitochondrial dysregulation, several studies in vitro have demonstrated ENs intestinal effects in specific cell lines, reporting its cytotoxicity and the activation of oxidative stress processes, even at low molecular concentrations [38]. The activity of three essential markers implicated in cellular response to oxidative stress was evaluated in vivo after ENs acute exposure. SOD1 is an antioxidant enzyme which regulates the superoxide levels from cytosol and mitochondrial intermembrane [39]. It has been observed that its deletion is related to ROS production and the reduction of antioxidant enzymes activities in colon tissues, agreeing with the downregulation observed in the present research [40]. Similarly, it has been observed that the higher activity of the key catalytic enzyme GPx1 promote the progression of distinct types of cancer, including colon cancer, which is in line with the overexpression observed after the highest dose of ENs mixture employed in female rats [41,42] As regard the intestinal inflammation modulator Hmox1, it has been shown to inhibit the activation of apoptotic pathways in different cell lines but in this case, did not show significant changes in its expression [43]. 

The homeostasis of the intestinal barrier is also disturbed by an imbalance that occurs between pro- and anti-apoptotic genes. In particular, it has been demonstrated that the p53 gene is involved in the initiation of the apoptosis mechanisms of the cellular cycle and its overexpression has been associated with different types of intestinal cancer [44,45]. Moreover, tumor suppressor p53 regulates the anti-apoptotic (Bcl2) and pro-apoptotic (Bax) members of the Bcl-2 proteins family, which control apoptosis by monitoring mitochondrial outer membrane permeabilization [46]. In this study, p53 and Bax genes were upregulated after ENs treatment, whereas on the contrary, anti-apoptotic Bcl-2 did not show significant changes, suggesting apoptosis activation. 

As a consequence of the development of oxidative-stress-induced cellular damage and apoptosis, the onset of inflammation and structural disfunction can successively occur [47]. The activity of the structural protein occludin, for instance, is related to intestinal epithelial disorders and abnormal secretory function [48]. Its upregulation in intestinal cells was implicated in the decrease of gut permeability in accordance with this result, hinting the activation of defense mechanisms [49]. 

## 5. Conclusions

ENs generated toxic responses in rat tissues at the mitochondrial level at the medium and high concentrations employed, more evident for MT-ND1 and MT-COX1 in all organs analyzed. Furthermore, the activation of oxidative stress and pro-apoptotic genes was shown in lower intestine tissues but not confirmed by inflammatory cytokine activity Il-1β. At the structural level, changes in the epithelial barrier have been observed, suggesting the decrease of barrier permeability. In conclusion, the results obtained in this study suggest that ENs could play a role in mitochondrial disorders and intestinal acute toxicity. However, there is a need of further investigation in ENs long-term exposure in order to survey their possible chronic effect on animals.

## Figures and Tables

**Table 1:** Target organ samples: control (C), medium dose (M), high dose (H) extracted RNA concentration (ng/μL), RNA purity ratios 260/280 and 260/230.

	Sample	RNA (ng/μL)	260/280	260/230
Stomach	C1	738.4	1.93	2.07
	C2	370.8	1.90	2.26
	C3	246.7	1.85	1.95
	C4	1051.2	2.01	2.15
	M1	546.4	1.89	1.76
	M2	647.1	1.96	2.07
	M3	923.2	1.95	2.10
	M4	1003.8	1.98	2.21
	M5	1312.1	1.96	2.07
	H1	607.8	1.92	2.08
	H2	1177.1	1.99	2.21
	H3	1388.9	2.02	2.01
	H4	652.0	1.92	2.08
	H5	1259.3	1.99	2.21
Liver	C1	664.1	2.10	2.24
	C2	695.8	2.10	2.20
	C3	493.9	2.07	2.10
	C4	638.0	2.11	2.28
	M1	914.3	2.16	2.24
	M2	2199.2	2.12	2.05
	M3	1613.9	2.17	2.22
	M4	1592.0	2.15	2.14
	M5	1340.0	2.18	2.14
	H1	1875.7	2.13	2.17
	H2	1017.6	2.15	2.32
	H3	1199.6	2.16	2.35
	H4	1502.6	2.16	2.31
	H5	1334.5	2.15	2.31
Kidneys	C1	1197.6	2.07	2.27
	C2	2359.5	2.01	2.16
	C3	1550.3	2.12	2.14
	C4	1615.1	2.1	2.25
	M1	1995.0	2.10	2.18
	M2	1585.0	2.07	2.26
	M3	1606.0	2.07	2.22
	M4	1211.6	2.11	2.16
	M5	600.8	2.06	2.26
	H1	329.6	2.06	2.25
	H2	247.4	2.11	2.30
	H3	715.7	2.10	2.15
	H4	615.0	2.10	2.15
	H5	2407.3	2.12	2.25
Colon	C1	1728.0	2.16	2.20
	C2	1581.0	2.18	2.20
	C3	759.0	2.17	2.22
	C4	2306	2.10	2.14
	M1	1838.0	2.15	2.20
	M2	1891.0	2.14	2.17
	M3	1407.0	2.16	2.23
	M4	1360.0	2.18	2.23
	M5	1619.0	2.17	2.19
	H1	1961.0	2.17	2.19
	H2	1736.0	2.16	2.19
	H3	838.0	2.17	2.21
	H4	1084.0	2.17	2.21
	H5	859.0	2.16	2.20

**Table 2:** Gene symbol, forward (F) and reverse (R) primers, efficiency and linearity of the selected genes plus reference genes β−actin and 18S rRNA.

Gene	Sequence	Efficiency	Linearity
MT-ND1	F: CGAGCTCCCTTCGACTTAAC R: GAATAGGGCGAATGGTCCTG	101.806	0.991
Sdha	F: GACGATCTCTGCGGTATGAC R: TCGGTGTATGGACCCATCTT	107.658	0.975
MT-COX1	F: GCTGGAGCATCCGTAGATTT R: ATTGGGTTATAGCAGGGGGT	106.425	0.985
ATP5	F: GTGATGTGTCCGCCTACATT R: ACAAGCCCACATTAATGGCA	107.423	0.978
SOD1	F: ACACAAGGCTGTACCACTGC R: CCACATTGCCCAGGTCTCC	124.573	0.991
GPx1	F: GTCCACCGTGTATGCCTTCTCC R: TCTCCTGATGTCCGAACTGATTGC	105.269	0.990
Hmox1	F: CACGCATATACCCGCTACCT R: AAGGCGGTCTTAGCCTCTTC	146.284	0.981
Bcl2	F: ACTGAGTACCTGAACCGGCATC R: GGAGAAATCAAACAGAGGTCGC	148.237	0.990
Bax	F: AAGAAGCTGAGCGAGTGTCT R: CAAAGATGGTCACTGTCTGC	117.003	0.981
p53	F: GTTCCGAGAGCTGAATGAGG R: TTTTATGGCGGGACGTAGAC	111.153	0.990
Nf-κB	F: CTTCTCGGAGTCCCTCACTG R: CCAATAGCAGCTGGAAAAGC	102.480	0.996
Il-1β	F: CTTGTCGAGAATGGGCAGTCT R: TGTGCCACGGTTTTCTTATGG	105.269	0.990
Occludin	F: AGTACATGGCTGCTGCTGATG R: CCCACCATCCTCTTGATGTGT	143.830	0.990
β-actin	F: AACCTTCTTGCAGCTCCTCCG R: CCATACCCACCATCACACCCT	94.242	0.996
18S rRNA	F: GAGCGTGTGATCACCATCAT R: TCCTTCACGTCCTTCTGTCT	105.487	0.979

## References

[1] Gil L., Ruiz P., Font G., Manyes L. (2016). An overview of the applications of hazards analysis and critical control point (HACCP) system to mycotoxins. Revista Toxicol..

[2] Escrivá L., Font G., Manyes L. (2015). In vivo toxicity studies of fusarium mycotoxins in the last decade: A review. Food Chem. Toxicol..

[3] Stanciu O., Juan C., Miere D., Loghin F., Mañes J. (2017). Occurrence and co-occurrence of Fusarium mycotoxins in wheat grains and wheat flour from Romania. Food Control.

[4] Fraeyman S., Meyer E., Devreese M., Antonissen G., Demeyere K., Haesebrouck F., Croubels S. (2018). Comparative in vitro cytotoxicity of the emerging Fusarium mycotoxins beauvericin and enniatins to porcine intestinal epithelial cells. Food Chem. Toxicol..

[5] Prosperini A., Berrada H., Ruiz M.J., Caloni F., Coccini T., Spicer L.J., Perego M.C., Lafranconi A. (2017). A Review of the Mycotoxin Enniatin B. Front. Public Health.

[6] Jonsson M. The Toxicity of Fusarium Mycotoxins Enniatin and Moniliformin. 2017. https://www.ruokavirasto.fi/globalassets/yhteisot/tieteellinen-tutkimus/vaitoskirjat/2017-jonsson.pdf.

[7] Gruber-Dorninger C., Novak B., Nagl V., Berthiller F. (2017). Emerging Mycotoxins: Beyond Traditionally Determined Food Contaminants. J. Agric. Food Chem..

[8] Fraeyman S., Croubels S., Devreese M., Antonissen G. (2017). Emerging Fusarium and Alternaria Mycotoxins: Occurrence, Toxicity and Toxicokinetics. Toxins.

[9] Maranghi F., Tassinari R., Narciso L., Tait S., Rocca C.L., Felice G.D., Butteroni C., Corinti S., Barletta B., Cordelli E. (2018). In vivo toxicity and genotoxicity of beauvericin and enniatins. Combined approach to study in vivo toxicity and genotoxicity of mycotoxins beauvericin (BEA) and enniatin B (ENNB). EFSA Supporting Publ..

[10] Cimbalo A., Alonso-Garrido M., Font G., Manyes L. (2020). Toxicity of mycotoxins in vivo on vertebrate organisms: A review. Food Chem. Toxicol..

[11] Jonsson M., Jestoi M., Anthoni M., Welling A., Loivamaa I., Hallikainen V., Kankainen M., Lysøe E., Koivisto P., Peltonen K. (2016). Fusarium mycotoxin enniatin B: Cytotoxic effects and changes in gene expression profile. Toxicol. In Vitro.

[12] Juan-García A., Manyes L., Ruiz M., Font G. (2013). Applications of flow cytometry to toxicological mycotoxin effects in cultured mammalian cells: A review. Food Chem. Toxicol..

[13] Alonso-Garrido M., Escrivá L., Manyes L., Font G. (2018). Enniatin B induces expression changes in the electron transport chain pathway related genes in lymphoblastic T-cell line. Food Chem. Toxicol..

[14] Alonso-Garrido M., Tedeschi P., Maietti A., Font G., Marchetti N., Manyes L. (2020). Mitochondrial transcriptional study of the effect of aflatoxins, enniatins and carotenoids in vitro in a blood brain barrier model. Food Chem. Toxicol..

[15] Alonso-Garrido M., Manyes L., Pralea I.E., Iuga C.A. (2020). Mitochondrial proteomics profile points oxidative phosphorylation as main target for beauvericin and enniatin B mixture. Food Chem. Toxicol..

[16] Bustin S.A., Benes V., Garson J.A., Hellemans J., Huggett J., Kubista M., Mueller R., Nolan T., Pfaffl M.W., Shipley G.L. (2009). The MIQE Guidelines: Minimum Information for Publication of Quantitative Real-Time PCR Experiments. Clin. Chem..

[17] Cimbalo A., Frangiamone M., Juan C., Font G., Lozano M., Manyes L. (2021). Proteomics evaluation of enniatins acute toxicity in rat liver. Food Chem. Toxicol..

[18] Iommarini L., Ghelli A., Tropeano C.V., Kurelac I., Leone G., Vidoni S., Lombes A., Zeviani M., Gasparre G., Porcelli A.M. (2018). Unravelling the effects of the mutation m. 3571insC/MT-ND1 on respiratory complexes structural organization. Int. J. Mol. Sci..

[19] Garcia-Ruiz I., Fernández-Moreira D., Solis-Munoz P., Rodriguez-Juan C., Diaz-Sanjuan T., Muñoz-Yagüe T., Solis-Herruzo J.A. (2010). Mitochondrial complex I subunits are decreased in murine nonalcoholic fatty liver disease: Implication of peroxynitrite. J. Proteome Res..

[20] Guo H., Bi X., Zhou P., Zhu S., Ding W. (2017). NLRP3 deficiency attenuates renal fibrosis and ameliorates mitochondrial dysfunction in a mouse unilateral ureteral obstruction model of chronic kidney disease. Mediat. Inflamm..

[21] Wallace L., Mehrabi S., Bacanamwo M., Yao X., Aikhionbare F.O. (2016). Expression of mitochondrial genes MT-ND1, MT-ND6, MT-CYB, MT-COI, MT-ATP6, and 12S/MT-RNR1 in colorectal adenopolyps. Tumor Biol..

[22] Huang S., Millar A.H. (2013). Succinate dehydrogenase: The complex roles of a simple enzyme. Curr. Opin. Plant Biol..

[23] Miettinen M., Lasota J. (2014). Succinate dehydrogenase deficient gastrointestinal stromal tumors (GISTs)–a review. Int. J. Biochem. Cell Biol..

[24] Boikos S.A., Pappo A.S., Killian J.K., LaQuaglia M.P., Weldon C.B., George S., Trent J.C., Von Mehren M., Wright J.A., Schiffman J.D. (2016). Molecular subtypes of KIT/PDGFRA wild-type gastrointestinal stromal tumors: A report from the National Institutes of Health Gastrointestinal Stromal Tumor Clinic. JAMA Oncol..

[25] Kamai T., Higashi S., Murakami S., Arai K., Namatame T., Kijima T., Abe H., Jamiyan T., Ishida K., Shirataki H. Single nucleotide variants of succinate dehydrogenase A gene in renal cell carcinoma. Cancer Sci..

[26] Abdulhag U.N., Soiferman D., Schueler-Furman O., Miller C., Shaag A., Elpeleg O., Edvardson S., Saada A. (2015). Mitochondrial complex IV deficiency, caused by mutated COX6B1, is associated with encephalomyopathy, hydrocephalus and cardiomyopathy. Eur. J. Hum. Genet..

[27] Jimenez P., Piazuelo E., Cebrian C., Ortego J., Strunk M., Garcia-Gonzalez M.A., Santander S., Alcedo J., Lanas A. (2010). Prostaglandin EP2 receptor expression is increased in Barrett’s oesophagus and oesophageal adenocarcinoma. Aliment. Pharmacol. Ther..

[28] Cathcart M., O’Byrne K.J., Reynolds J.V., O’Sullivan J., Pidgeon G.P. (2012). COX-derived prostanoid pathways in gastrointestinal cancer development and progression: Novel targets for prevention and intervention. Biochim. Biophys. Acta BBA Rev. Cancer.

[29] Martín-Sanz P., Mayoral R., Casado M., Boscá L. (2010). COX-2 in liver, from regeneration to hepatocarcinogenesis: What we have learned from animal models?. World J. Gastroenterol. WJG.

[30] Xiao J., Liong E.C., Huang H., On Tse W., Lau K.S., Pan J., Nanji A.A., Fung M.L., Xing F., Tipoe G.L. (2015). Cyclooxygenase-1 serves a vital hepato-protective function in chemically induced acute liver injury. Toxicol. Sci..

[31] Harris R.C. (2013). Physiologic and pathophysiologic roles of cyclooxygenase-2 in the kidney. Trans. Am. Clin. Climatol. Assoc..

[32] Sarkar S., Mukherjee S., Chattopadhyay A., Bhattacharya S. (2017). Differential modulation of cellular antioxidant status in zebrafish liver and kidney exposed to low dose arsenic trioxide. Ecotoxicol. Environ. Saf..

[33] Jonckheere A.I., Smeitink J.A., Rodenburg R.J. (2012). Mitochondrial ATP synthase: Architecture, function and pathology. J. Inherit. Metab. Dis..

[34] Aiyar R.S., Bohnert M., Duvezin-Caubet S., Voisset C., Gagneur J., Fritsch E.S., Couplan E., Von Der Malsburg K., Funaya C., Soubigou F. (2014). Mitochondrial protein sorting as a therapeutic target for ATP synthase disorders. Nat. Commun..

[35] Zhang J., Schmidt C.J., Lamont S.J. (2018). Distinct genes and pathways associated with transcriptome differences in early cardiac development between fast-and slow-growing broilers. PLoS ONE.

[36] Feichtinger R.G., Schäfer G., Seifarth C., Mayr J.A., Kofler B., Klocker H. (2018). Reduced levels of ATP synthase subunit ATP5F1A correlate with earlier-onset prostate cancer. Oxidative Med. Cell. Longev..

[37] Xu G., Li J.Y. (2016). ATP5A1 and ATP5B are highly expressed in glioblastoma tumor cells and endothelial cells of microvascular proliferation. J. Neurooncol..

[38] Bertero A., Fossati P., Tedesco D.E.A., Caloni F. (2020). Beauvericin and Enniatins: In Vitro Intestinal Effects. Toxins.

[39] Eleutherio E.C.A., Magalhães R.S.S., de Araújo Brasil A., Neto J.R.M., de Holanda Paranhos L. (2021). SOD1, more than just an antioxidant. Arch. Biochem. Biophys..

[40] Gonzalez-Menendez P., Hevia D., Alonso-Arias R., Alvarez-Artime A., Rodriguez-Garcia A., Kinet S., Gonzalez-Pola I., Taylor N., Mayo J.C., Sainz R.M. (2018). GLUT1 protects prostate cancer cells from glucose deprivation-induced oxidative stress. Redox Biol..

[41] Gan X., Chen B., Shen Z., Liu Y., Li H., Xie X., Xu X., Li H., Huang Z., Chen J. (2014). High GPX1 expression promotes esophageal squamous cell carcinoma invasion, migration, proliferation and cisplatin-resistance but can be reduced by vitamin D. Int. J. Clin. Exp. Med..

[42] Wei R., Qiu H., Xu J., Mo J., Liu Y., Gui Y., Huang G., Zhang S., Yao H., Huang X. (2020). Expression and prognostic potential of GPX1 in human cancers based on data mining. Ann. Transl. Med..

[43] Ribeiro J., Malta M., Galaghar A., Silva F., Afonso L.P., Medeiros R., Sousa H. (2017). P53 deregulation in Epstein-Barr virus-associated gastric cancer. Cancer Lett..

[44] Ryter S.W. (2021). Heme Oxgenase-1, a Cardinal Modulator of Regulated Cell Death and Inflammation. Cells.

[45] Kim K.W., Kim N., Choi Y., Kim W.S., Yoon H., Shin C.M., Park Y.S., Lee D.H., Park Y.S., Ahn S. (2021). Different effects of p53 protein overexpression on the survival of gastric cancer patients according to Lauren histologic classification: A retrospective study. Gastric Cancer.

[46] Dashzeveg N., Yoshida K. (2015). Cell death decision by p53 via control of the mitochondrial membrane. Cancer Lett..

[47] Netea M.G., Balkwill F., Chonchol M., Cominelli F., Donath M.Y., Giamarellos-Bourboulis E.J., Golenbock D., Gresnigt M.S., Heneka M.T., Hoffman H.M. (2017). A guiding map for inflammation. Nat. Immunol..

[48] Zhao X., Zeng H., Lei L., Tong X., Yang L., Yang Y., Li S., Zhou Y., Luo L., Huang J. (2021). Tight junctions and their regulation by non-coding RNAs. Int. J. Biol. Sci..

[49] Zhou Z., Bian C., Luo Z., Guille C., Ogunrinde E., Wu J., Zhao M., Fitting S., Kamen D.L., Oates J.C. (2019). Progesterone decreases gut permeability through upregulating occludin expression in primary human gut tissues and Caco-2 cells. Sci. Rep..

